# Directional Darwinian Selection in proteins

**DOI:** 10.1186/1471-2105-14-S13-S6

**Published:** 2013-10-01

**Authors:** David A McClellan

**Affiliations:** 1 Department of Biology, University of Arkansas-Fort Smith, Fort Smith, AR 72913, USA; 2 Bigelow Laboratory for Ocean Sciences, East Boothbay, ME 04544, USA

## Abstract

**Background:**

Molecular evolution is a very active field of research, with several complementary approaches, including *dN*/*dS*, HON90, MM01, and others. Each has documented strengths and weaknesses, and no one approach provides a clear picture of how natural selection works at the molecular level. The purpose of this work is to present a simple new method that uses quantitative amino acid properties to identify and characterize *directional *selection in proteins.

**Methods:**

Inferred amino acid replacements are viewed through the prism of a single physicochemical property to determine the amount and direction of change caused by each replacement. This allows the calculation of the probability that the mean change in the single property associated with the amino acid replacements is equal to zero (H_0_: *μ *= 0; i.e., no net change) using a simple two-tailed t-test.

**Results:**

Example data from calanoid and cyclopoid copepod cytochrome oxidase subunit I sequence pairs are presented to demonstrate how directional selection may be linked to major shifts in adaptive zones, and that convergent evolution at the whole organism level may be the result of convergent protein adaptations.

**Conclusions:**

Rather than replace previous methods, this new method further complements existing methods to provide a holistic glimpse of how natural selection shapes protein structure and function over evolutionary time.

## Background

Natural selection, as first outlined by Charles Darwin, acts on phenotypes:

"She [natural selection] can act on every internal organ, on every shade of constitutional difference, on the whole machinery of life...Under nature, the slightest difference of structure or constitution may well turn the nicely-balanced scale in the struggle for life, and so be preserved...It may be said that natural selection is daily and hourly scrutinizing, throughout the world, every variation, even the slightest; rejecting that which is bad, preserving and adding up all that is good..." [1].

We can think of natural selection as collecting adaptations that optimize an organism's survival, reproductive success, and fecundity in a given environment or habitat. As Darwin explicitly states above, this process is not limited to the phenotypes of the whole organism; it works on "every variation, even the slightest." Although we sometimes think of proteins in this way, there currently is not a consistently reliable method for identifying and characterizing the evolution of protein phenotypes. This being stated, science is currently faced with the challenge of assessing the impact that anthropogenic climate change is likely to have with potentially catastrophic effects at the base of the food chain on the molecular level. The scientific community's efforts to produce realistic solutions to the big problems associated with climate change will be greatly enhanced by the development of more robust analytical methods for comprehensively characterizing the effects of natural selection in terms of the biochemistry and physics of protein structure, function, and interactions.

Several statistical methods for identifying and characterizing selection at the molecular level have been proposed since the genetic code was determined in the 1960s. Of these, three classes of methods dominate the literature. The first, and most significant, is the family of methods that implements one of many variations of the nonsynonymous-to-synonymous substitution rate ratio, or *dN*/*dS *(e.g., [2-15]). Briefly, this approach compares the rate of nonsynonymous (*dN*), or amino acid changing, nucleotide substitutions with the rate of synonymous (*dS*), or silent, nucleotide substitution. When *dN *is significantly greater than *dS*, the system is said to have been influenced by positive selection, when *dN *= *dS*, the system is said to be neutral, and *dN *<*dS *indicates negative selection. This family of methods is broadly accepted and implemented, and has enjoyed a great deal of success. This simple model, however, has several shortcomings, including problems with the underlying assumptions (e.g., [16-19]) and difficulties accurately estimating rates when divergences are very small and great, and is not sensitive enough to detect natural selection in some protein coding genes when it is known to have taken place (e.g., [20-22]).

As a reaction to weaknesses of *dN*/*dS *approaches, Hughes et al. [23] presented a similar approach (hereafter referred to as HON90) that compares proportions of conservative (*p_NC_*) and radical (*p_NR_*) amino acid replacement in terms of *qualitative *properties of amino acids to detect selection promoting charge profile diversity in class I MHC proteins. When *p_NR _> p_NC_*, the property of interest is said to have changed more than would be expected under random conditions. The Hughes et al. study [23] was the first to implement amino acid properties--in this case charge, polarity, hydrophobicity, and volume--to identify selection at the protein level. From a conceptual standpoint, this approach presented a method to assess patterns of amino acid replacement using the phenotypes of proteins, thus providing an avenue of analysis more consistent with Darwin's original definition of natural selection. This protein-level phenotypic approach has since been implemented several times and has yielded encouraging results (e.g., [24-28]).

In an effort to tap into the wealth of information afforded by the implementation of *quantitative *amino acid properties, researchers have expanded upon the HON90 approach in a number of ways, including the use of a spectrum of magnitude categories [18,29], a sliding window [30], accuracy benchmarking [31], and potential uses for characterizing single amino acid replacements [32]. These approaches (hereafter referred to as MM01 methods) take the underlying pattern of nucleotide composition into account. The collective identity of properties that individually yield positive statistical results provides clues that link specific genetic variants to selective advantages and disadvantages afforded by known changes in ambient environment [18,33,34]. The robustness of the results yielded by MM01 approaches is greatly enhanced by the wealth of information emerging from crystallography and magnetic resonance experiments that determine protein structures with a high degree of precision and accuracy. Results localized to protein regions of known structures and functions provide evidence useful for comprehensively characterizing protein function evolution [30,34-38].

### Existing solutions fall short

Reconstructing evolutionary events at the molecular level and diagnosing them in terms of natural selection has been an extraordinary challenge. Each individual point mutation carries with it just a small quantum of information. Patterns emerge as these quanta accumulate over evolutionary time. Oversimplifying models used to assess patterns of evolutionary information emerging from molecular data results in a net loss of analytical yield. Overparameterizing models has the opposite effect, producing more detail than can be realistically supported by the data. When studying a phenomenon as nuanced and multifaceted as molecular evolution, striking a happy medium between oversimplification and overparameterization is extremely difficult. Researchers want to squeeze every ounce of information from their data without seeing patterns that are not really there.

It is not surprising that *dN*/*dS *approaches sometimes ignore signs of natural selection that other methods pick up. *dN*/*dS *is a simple method, with several documented limitations [16-22]. The HON90 approach takes a step forward by incorporating amino acid properties, but the number of qualitative properties is limited; if the evolution of protein-coding gene sequences cannot be linked to charge, polarity, hydrophobicity, volume, or just a handful of other properties, negative results will be produced. Although these properties are important in terms of protein function, they likely are not the only properties affected by natural selection.

MM01 approaches present several advantages over *dN*/*dS *and HON90 methods (e.g., [18,30,35,36,39-45]). However, in an effort to force a greater information yield from the data, this method may be parameterizing systems to the point that accuracy suffers [31,32,46]. Clearly, this third class of approaches performs better in some circumstances, such as when divergences are very great and rates of synonymous change are underestimated [18], or when divergences are very small and synonymous changes have not had time to accumulate [32].

## Methods

The high frequency with which new genomes and metagenomes are being produced also suggests that a method with the potential for high-throughput that does not require information from underlying nucleotides is needed. Gene annotations produce a huge number of BLAST results [47,48]. Many of these are in the form of aligned protein, and not nucleotide, sequences. None of the methods outlined above are capable of screening this type of information for signs of molecular adaptation and cannot be utilized for studying adaptive changes at the genomic or metagenomic levels.

There is at least one aspect of physicochemical evolution that has been largely overlooked: *the direction of selection*. One exception is the study by Merritt and Quattro [27]. They identified a case in which positive selection resulted in a biased accumulation of negatively charged amino acids after a gene duplication event. However, changes in charge are generally rare in protein evolution [27,49,50] and, as discussed, the possible qualitative properties to test in the way Merritt and Quattro present are few in number. Testing for directional shifts in *quantitative *properties, of which there are now several hundred catalogued in the Japanese database AAindex [51], will allow for more comprehensive exploration of property space, and will likely result in a more clearly resolved vision of how proteins adapt to the specific needs of organisms as they evolve in changing habitats. Such a new method, when coupled with existing methods, will provide a full set of analytical tools for identifying and characterizing molecular adaptation in a biologically meaningful way.

A method similar to that presented by Merritt and Quattro [27] that allows for the implementation of quantitative physicochemical amino acid properties will require a different statistical test. Inferred amino acid replacements will be viewed through the prism of a single physicochemical property to determine the amount and direction of change caused by each replacement. This will allow the calculation of the probability that the mean change in the single property associated with the amino acid replacements is equal to zero (H_0_: *μ *= 0; i.e., no net change) using a simple two-tailed t-test.

The novel aspect of this new method is its criterion. It evaluates amino acid replacements multi-dimensionally across a great number of physicochemical amino acid properties, and identifies instances of several amino acid replacements across several sites, evolving across phylogenetic space in the same physicochemical direction in a single dimension of property space. This approach makes the study of molecular evolution more applicable to studies that link patterns of amino acid replacement with environmental changes through time or space. A directional approach represents a return to the fundamental concept that selection affects phenotypes, while at the same time simplifying implementation. By so doing, interpretation of results will be less ambiguous.

The new method begins with a list of amino acid differences that includes the location of each in the context of the linear sequence of nucleotide codons and/or amino acids, depending on the input data. This list can be generated using an ancestral character-state reconstruction algorithm (such as *codeml *[52]) if the input is a multiple sequence alignment and a phylogenetic structure, or by pairwise comparison if the input is the results of a BLAST search [47,48]. From this list, the magnitude and direction (i.e., an increase or a decrease) of change in each amino acid property under consideration is inferred. A simple two-tailed t-test may be performed for each property to statistically evaluate the null hypothesis that the net change is equal to zero. The value of the t*-*test statistic is calculated using simple established equations:

(1) t=X¯sX¯N

(2) $$s_{\bar{X}}=\sqrt{\frac{\Sigma X_{i}^{2}-\frac{{\left(\Sigma X_{i}\right)}^{2}}{N}}{N-1}}$$

Here $X_{i}$ is the value of the change in amino acid property for each inferred amino acid difference,  , and  is the total number of amino acid differences. In the example below (Table 1), the value of $X_{i}$ for the difference at residue site 82 is +7.0, while the value of  is 15.

**Table 1:** Directional selection analysis of *Pan *and *Homo *SAGE1

Residue	*Pan*	*Homo*	Δ Hydropathy
82	Cys	Arg	+7.0
92	Val	Ala	+2.4
160	Arg	His	-1.3
450	Gln	Arg	+1.0
507	Asp	Val	-7.7
523	Ser	Thr	-0.1
563	Val	Ala	+2.4
582	Val	Asp	+7.7
605	Phe	Leu	-1.0
672	Ala	Thr	+2.5
675	Ser	Asn	+2.7
694	Thr	Ala	-2.5
754	Cys	Arg	+7.0
802	Val	Ala	+2.4
805	Leu	Ser	+4.6
		**Net Change =**	+27.1

Residue sites of the 15 *Pan troglodytes *and *Homo sapiens *SAGE1 (inferred from XM_001137139 and NM_018666, respectively) protein differences, with character states and net change in hydropathy [54].

The data may be partitioned in several different ways: A sliding window may be implemented to evaluate potential clustering of unidirectional changes; known or estimated secondary structures may be used to group amino acid differences according to the structural components of the protein; the range of amino acid sites corresponding to the functional domains of the protein may be used. How the data are partitioned is largely contingent on the scientific question, the amount and type of differences in the data, and the amount of supporting structure and function information available. In each case, care must be taken to partition the data in biologically meaningful ways that test specific hypotheses.

There are over 500 physicochemical amino acid properties on the AAindex database [51] available to assess amino acid differences. For the purposes of this study, the 25 properties in Table 2 were chosen to be representative of the breadth of amino acid property space. These properties describe aspects of proteins that are important to overall structure (e.g., molecular size, hydrophobicity, secondary structures) and function (e.g., ionization, non-bonded energy, solvent accessibility); properties that can potentially be affected by natural selection.

**Table 2:** Quantitative physicochemical amino acid properties

Category/Property	Symbol	Reference	Description*^a^*
**Hydrophobicity**			
Hydropathy	*h*	[54]	The hydrophilic and hydrophobic inclinations of a given residue side chain in terms of transfer of free energy.
Surrounding hydrophobicity	*H_p_*	[55]	The average sum of residue hydrophobic indices within an optimum sphere of 8 Å radius around a residue in protein crystals (kcal/mol).
Thermodynamic transfer hydrophobicity	*H_t_*	[56]	The experimental values of Noazaki & Tanford [57] combined with values of Zimmerman et al. [58] adjusted to the same scale (kcal/mol).
**Ionization Constants**			
Equilibrium constant	*pK'*	[56]	The ionizable character of the carboxyl group (pH units).
Isoelectric point	*pH_i_*	[58]	The isoionic point of the free amino acid, including the ionizable character of the entire residue (pH units).
**Molecular Size & Composition**			
Bulkiness	*B_l_*	[58]	The ratio of the side chain volume to length (i.e., the average cross section of the chain) (Å^2^).
Composition	*c*	[59]	The atomic weight ratio of the non-carbon elements in the end groups or rings to carbons in the side chain.
Molecular weight	*M_w_*	[60]	The mass of the atoms constituting the residue.
Partial specific volume	*V*^0^	[61]	The reciprocal of density (m^3 ^mol^-1 ^× 10^-6^).
**Non-bonded Energy**			
Long range energy	*E_l_*	[62]	The energy between two amino acids separated further than 10 residues (i.e., due to electrostatic and Van der Waals forces) (kcal/mol).
Short and medium range energy	*E_sm_*	[62]	The sum of the energy between 1) main chain atoms of a residue and its own side chain atoms, and 2) two residues located within 10 residues along the chain (kcal/mol).
Total non-bonded energy	*E_t_*	[62]	Sum of average short, medium, and long range non-bonded energies (kcal/mol).
**Polarity & Polarizability**			
Polar requirement	*P_r_*	[63]	The slope of the line regressing log *R_M _*and the mol fraction of water in the pyrimidine-water solvent (*R_M _= *1/*R_F _*- 1, where *R_F _*is the chromatographic index [58]).
Polarity	*p*	[59]	The average of *P_r _*and *P_A _*(*P_A _*= 13.66 - 14.85*R_F_*).
Refractive index	μ	[55]	The measure of the polarizability of a residue (i.e., the reciprocal measure of its electrical stability under an external field).
**Secondary Structure**			
Alpha-helical tendency	*P_α_*	[64]	The average intrinsic property of a residue to adopt an alpha-helical conformation.
Beta-structure tendency	*P_β_*	[64]	The average intrinsic property of a residue to adopt a beta-sheet conformation.
Coil tendency	*P_c_*	[65]	A measure of the tendency that a particular residue will be found in a coil secondary structure.
Helical contact area	*C_a_*	[66]	The maximum area loss that could occur in going from an isolated α-helix to a fully buried environment in the complex (Å^2^).
Turn tendency	*P_t_*	[64]	The average intrinsic property of a residue to adopt a beta-turn conformation.
**Solvent Accessibility**			
Solvent accessibility reduction ratio	*R_a_*	[67]	The ratio of solvent accessibility: the solvent accessibility of a residue in a hypothetically extanded state over its accessibility in a native folded protein.
**Tertiary Structure**			
Average number of surrounding residues	*N_a_*	[67]	The average number of residues surrounding a residue within the effective distance of influence.
Buriedness	*B_r_*	[61]	The average propensity of a residue to be found in the interior of a protein.
Compressibility	*K*^0^	[61]	The relative increase in the volume of the system per unit decrease in pressure (m^3 ^mol^-1 ^P_a_^-1 ^× 10^-15^).
Mean rms fluctuational displacement	*F*	[68]	The relationship between the average amount of root-mean-square displacement of a residue and its distance from the centroid of the protein (Å).

*^a ^*Properties without defined units are dimensionless.

Twenty-five amino acid properties representative of the breadth of amino acid property space.

Together, these four complementary methods will enable more robust evaluation of data than is possible with any single method: *dN*/*dS *methods focus on patterns of nucleotide substitution; HON90 looks at phenotypic patterns across amino acid changes; MM01 methods emphasize patterns among the most radical changes; the new method detects localized directional shifts in protein phenotypes. Furthermore, certain methods are able to more easily accommodate different data types. All of the methods can assess multiple protein-coding nucleotide sequence alignments with an accompanying phylogenetic structure, but *dN*/*dS *methods, for example, are unable to evaluate *blastp *output because there is no way to estimate the rate of synonymous change in the encoding DNA sequences from aligned amino acid sequences. The new directional selection method will easily accept *blastp *output because it does not require information about the underlying pattern of nucleotides or the governing genetic code.

## Results and discussion

### Directional selection linked to Habitat Shifts

Several marine and freshwater calanoid copepod cytochrome oxidase subunit I (COI) sequence pairs were considered. The first approximately 650 nucleotides of the cytochrome oxidase subunit 1 coding region for each were obtained from the Barcode of Life Database (http://www.barcodinglife.com) and evaluated using the directional selection approach. The comparison of *Calanus hyperboreus *(marine) and *Mastigodiaptomus montezumae *(freshwater) is representative (GenBank accession numbers FJ602504 and EU770508, respectively). Interestingly, the first 650 nucleotides encode all of the components of the first COI proton pump [53]. There are 11 amino acid differences within the first 215 amino acid residue sites for this species pair. These replacements have resulted in radical changes in several physicochemical properties. None of the properties were implicated in the proton output region of the protein (*p *< 0.05), but three properties affected the proton input region: one that describes hydrophobicity (*H_p_*), one for polarity (*P_r_*), and one for tertiary structure (*F*). Collectively, these properties, coupled with their direction of change, indicate that the proton input region became less hydrophobic, more polar, and more structural malleability during calanoid adaptation to freshwater, resulting in a more direct and less energetically expensive path for hydrogen ions to penetrate the membrane and enter the proton pump.

Several marine and freshwater cyclopoid copepod cytochrome oxidase subunit I (COI) sequence pairs were considered as well. Of these, the comparison of *Oithona similis *(marine) and *Thermocyclops inversus *(freshwater) is representative (GenBank accession numbers EU599544 and EU770551, respectively). There are 40 amino acid differences within the first 215 residue sites of COI for this species pair. Five properties yielded statistically significant directional results (*p *< 0.05) across the entire alignment, including *V*^0^, *P_r_*, *p*, μ, and *H_t_*. Like the calanoid data, the cyclopoid data failed to exhibit positive results in the proton output region. The proton input region, however, experienced significant directional change in 12 properties (Table 3). The identity of the properties and the direction of change were similar to the calanoid results, indicating a decrease in hydrophobicity (*h*, *H_p_*, *H_t_*), an increase in polarity (*P_r_*, *p*), and increased structural malleability (*N_a_*, *B_r_*, *F*), but cyclopoids also exhibited a decrease in molecular size (*B_l_*, *V*^0^) and total non-bonded energy (*E_t_*), and an increase in turn tendency (*P_t_*). Collectively, these results suggest an even more direct and less energetically expensive path for hydrogen ions to enter the proton pump than exhibited by the calanoids.

**Table 3:** Results of directional selection analysis of marine and freshwater copepod COI

	Calanoids		Cyclopoids	
	
Properties	Proton Input	Proton Output	Proton Input	Proton Output
**Hydrophobicity**				
* h*	**- - -**			
* H_p_*	**- -**		**- -**	
* H_t_*	**-**		**-**	
**Ionization Constants**				
* pK'*				
* pH_i_*				
**Molecular Size**				
* B_l_*	**- - -**			
* c*				
* M_w_*	**-**			
* V*^0^	**- - -**		**-**	
**Non-bonded Energy**				
* E_l_*	**-**			
* E_sm_*				
* E_t_*	**- -**			
**Polarity & Polarizability**				
* P_r_*	**+ + +**		**+ +**	
* p*	**+ + +**		**+**	
μ	**- -**		**-**	
**Secondary Structure**				
* P_α_*				
* P_β_*				
* P_c_*	**+**			
* C_a_*	**-**		**-**	
* P_t_*	**+ +**			
**Solvent Accessibility**				
* R_a_*				
**Tertiary Structure**				
* N_a_*	**- - -**			
* B_r_*	**- -**		**-**	
* K*^0^	**+**			
* F*	**+ + +**		**+ +**	

Properties among the 25 defined in Table 2 that experienced significant directional shifts as copepods evolved from being adapted to a marine habitat to life in freshwater ("**+**" indicates significant increases in the property, while "**-**" indicates significant decreases in the property; one symbol indicates significance at the α = 0.1 level, while two symbols indicate significance at the α = 0.05 level and three indicates α = 0.02 significance).

Interestingly, the calanoid and cyclopoid results appear parallel at the property level even though none of the specific sites affected were the same. To illustrate even the subtle parallel shifts in properties, Table 3 also includes those properties that yielded results at a lower significance (*p *= 0.1). Every property affected during calanoid adaptation to freshwater was also affected during cyclopoid adaptation to freshwater, and in the same direction. Cyclopoids had a greater number of affected properties likely due to a greater accumulation of amino acid replacements.

The discovery that these two lineages of copepods found parallel routes for COI functional adaptation is the most exciting conclusion of these results. These findings suggest that the amazing amount of convergence in the natural world may be the result of a limited number of alternative physicochemical strategies. This partially explains how independently evolving proteins can converge upon similar structures and functions when sequence identity remains low. Furthermore, the consistency of these results demonstrates how analyzing protein-coding genes in terms of changing protein phenotypes provides a link between the evolution of organisms and the influence of environmental variables, and hints at the actual causes of natural selection.

## Conclusions

The methods for identifying and characterizing natural selection at the molecular level, *dN*/*dS*, HON90, and MM01, use different aspects of the evolutionary information locked in protein-coding sequencing sequences. However, none of these methods are able to identify signs of adaptation in protein sequences without the aid of the underlying nucleotide information. A new method for identifying adaptation in either protein *or *protein-coding DNA sequences is presented. Collectively, the four methods will enable a more robust evaluation of existing data than is possible with any single method. Furthermore, the new directional selection method can tap the wealth of information in BLAST reports, like those emerging from genome and metagenome annotation efforts. It is likely that high-throughput analysis of annotation reports will provide a glimpse of the collective evolutionary forces that shape the morphologies and behaviors at the organismal level, especially as they evolve in a changing environment, providing a strong link between macroevolution and microevolution. Such a link will likely prove important to improving our understanding of how modern anthropogenic changes in global and local climates may be affecting vulnerable organisms over evolutionary time or at more accelerated rates.

## Competing interests

There are no competing interests with regard to this research.

## Authors' contributions

This research was performed by the sole author.

## Declarations

Funding for publication was provided by the Department of Biological Science at University of Arkansas-Fort Smith.

This article has been published as part of BMC Bioinformatics Volume 14 Supplement 13, 2013: Selected articles from the 9th Annual Biotechnology and Bioinformatics Symposium (BIOT 2012). The full contents of the supplement are available online at http://www.biomedcentral.com/bmcbioinformatics/supplements/14/S13
